# Improving the Solubilization and Bioavailability of Arbidol Hydrochloride by the Preparation of Binary and Ternary β-Cyclodextrin Complexes with Poloxamer 188

**DOI:** 10.3390/ph14050411

**Published:** 2021-04-26

**Authors:** Md. Khalid Anwer, Muzaffar Iqbal, Mohammad Muqtader Ahmed, Mohammed F. Aldawsari, Mohd Nazam Ansari, Essam Ezzeldin, Nasr Y. Khalil, Raisuddin Ali

**Affiliations:** 1 Department of Pharmaceutics, College of Pharmacy, Prince Sattam Bin Abdulaziz University, Alkharj 11942, Saudi Arabia; mkanwer2002@yahoo.co.in (M.K.A.); mo.ahmed@psau.edu.sa (M.M.A.); moh.aldawsari@psau.edu.sa (M.F.A.); 2 Department of Pharmaceutical Chemistry, College of Pharmacy, King Saud University, Riyadh P.O. Box 2457, Saudi Arabia; ezzeldin24@hotmail.com (E.E.); nasrykhalil@hotmail.com (N.Y.K.); 3 Bioavailability Unit, Central Laboratory, College of Pharmacy, King Saud University, Riyadh P.O. Box 2457, Saudi Arabia; 4 Department of Pharmacology, College of Pharmacy, Prince Sattam Bin Abdulaziz University, Alkharj 11942, Saudi Arabia; m.ansari@psau.edu.sa; 5 Department of Pharmaceutics and Central Lab, College of Pharmacy, King Saud University, Riyadh P.O. Box 2457, Saudi Arabia; ramohammad@ksu.edu.sa

**Keywords:** arbidol, β-cyclodextrin, poloxamer 188, ternary complex, solubilization, bioavailability

## Abstract

In the current study, the effect of poloxamer 188 on the complexation efficiency and dissolution of arbidol hydrochloride (ADL), a broad-spectrum antiviral agent, with β-cyclodextrin (β-CD) was investigated. Phase solubility studies confirmed a stoichiometry of a 1:1 ratio for both ADL:β-CD and ADL/β-CD with a 1% poloxamer 188 system with an AL type of phase solubility curve. The stability constants (K1:1) calculated from the AL type diagram were 550 M-1 and 2134 M-1 for AD:β-CD and ADL/β-CD with 1% poloxamer 188, respectively. The binary ADL/β-CD and ternary ADL/β-CD with 1% poloxamer 188 complexes were prepared by kneading and a solvent evaporation method and were characterized by aqueous solubility, FTIR, PXRD, DSC and SEM in vitro studies. The solubility (13.1 fold) and release of ADL were markedly improved in kneaded ternary ADL/β-CD with 1% poloxamer 188 (KDB). The binding affinity of ADL and β-CD was confirmed by ^1^H NMR and 2D ROSEY studies. The ternary complex (KDB) was further subjected for in vivo pharmacokinetic studies in rats and a significant improvement in the bioavailability (2.17 fold) was observed in comparison with pure ADL. Therefore, it can be concluded that the solubilization and bioavailability of ADL can be remarkably increased by ADL/β-CD complexation in the presence of a third component, poloxamer 188.

## 1. Introduction

Arbidol hydrochloride (ADL), also known as umifenovir, is a Russian-made potent broad-spectrum antiviral agent. ADL is used for prophylaxis as well as the treatment of human pulmonary diseases caused by influenza A and B viruses, hepatitis C and other human pathogenic respiratory viruses [1,2,3]. Since 2020, there has been a significant increase in research publications focusing on ADL due to its beneficial effects in coronavirus disease (COVID-19) patients. In clinical trials, ADL has been found to accelerate fever recovery and the virus clearance of respiratory specimens and also help shorten the length of hospital stays in COVID-19 patients with no major adverse effects [4,5]. ADL has shown broad-spectrum in vitro and in vivo antiviral activity against various types of enveloped or non-enveloped RNA or DNA viruses including respiratory viruses, hepatitis viruses and the encephalitis virus [6]. Recently, it has been reported that ADL could inhibit COVID-19 infection through interfering with the release of SARS-CoV-2 from intracellular vesicles [7]. 

Being indole derivative, ADL is hydrophobic in nature and is poorly soluble in aqueous media, which results in poor bioavailability in humans (40%) and rats (18.8%) [8,9]. Several efforts have been taken up previously to improve the water solubility of ADL including water soluble acrylamide polymers’ ADL complex formation [10] by dissolving ADL in hot glycerol at 23 °C before dilution with aqueous media [1] and also by improving the dissolution rate by a solid dispersion technique [11]. However, no pharmacokinetic nor metabolite studies were performed from this mode of administration. In addition, salt engineering techniques were also used to prepare the methanesulfonic salt [12] and salicylate anions form [13] of ADL in order to improve the aqueous solubility and bioavailability. The methanesulfonic salt of arbidol exhibited a relatively higher peak plasma concentration (Cmax) and an increased area under curve (AUC0-t) compared with a commercial product indicating the improved bioavailability of the drug; however, it was not statistically justified. Thus, a need arises to develop a water soluble formulation that allows a better dissolution and absorption and, consequently, improved bioavailability. With the improvement of ADL solubility, it would be possible to obtain formulations with low doses, a reduced dose frequency and better compliance access of the medicine.

Owing to their non-toxic nature and complexation ability, a cyclodextrins (CDs)-based drug delivery system is commonly employed as a promising platform to increase drug solubility and stability, which enhances the drug release profile [14]. In this context, the inclusion complexes of poorly water soluble drugs with α-cyclodextrin, β-cyclodextrin and γ-cyclodextrin are widely reported in the literature [15,16]. However, there are a few limitations for native CDs such as a small cavity size of α-cyclodextrin and γ-cyclodextrin, which are comparatively expensive. Therefore, β-cyclodextrin is the most commonly preferred for drug delivery applications [17].

Poloxamer 188 is an FDA (Food and Drug Administration) approved non-ionic block polymer composed of two hydrophilic side chains attached to a hydrophobic core and is frequently used for the solubility enhancement of poorly soluble drugs due to its excellent surfactant property [18,19,20]. Herein, we report the preparation and characterization of β-cyclodextrin-ADL (ADL/β-CD) complexes with 1% poloxamer 188 in order to improve the solubilization and bioavailability properties.

## 2. Results and Discussion

### 2.1. Phase Solubility Studies for ADL in Binary and Ternary Systems

**Figure 1:**
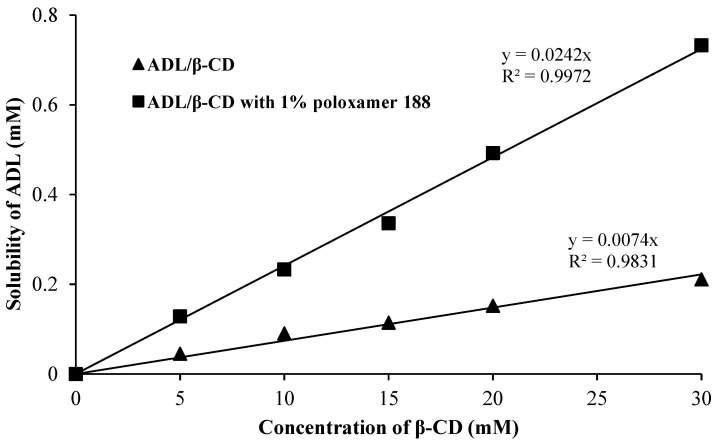
Phase solubility curves of ADL/β-CD and ADL/β-CD with 1% poloxamer 188.

The phase solubility diagrams of binary ADL/β-CD and ternary ADL/β-CD with 1% poloxamer 188 systems showed linear AL type solubility curves of ADL against an increasing concentration of β-CD and β-CD with 1% poloxamer 188 (Figure 1). According to Higuchi and Connors, an AL type curve with slope values less than 1 (linear drug-β-CD phase solubility diagram) suggested the formation of a 1:1 stoichiometric complex [21]. The data of apparent stability constants (Ks) and complexation efficiencies of the prepared complexes are shown in Table 1. The apparent stability constants (Ks) were obtained for ADL/β-CD and ADL/β-CD with 1% poloxamer 188 systems as 550 M^−1^ and 2134 M^−1^, respectively. The complexation efficiency (CE) was considered as more reliable data for complexation. The CE of ADL/β-CD and ADL/β-CD with 1% poloxamer 188 systems were found to be 0.0064 and 0.0250, respectively. It was revealed from the data that the addition of 1% poloxamer 188 to β-CD enhanced the interaction of ADL with β-CD by increasing the stability constant.

### 2.2. Aqueous Solubility Determination of the Solid Complexes

**Figure 2:**
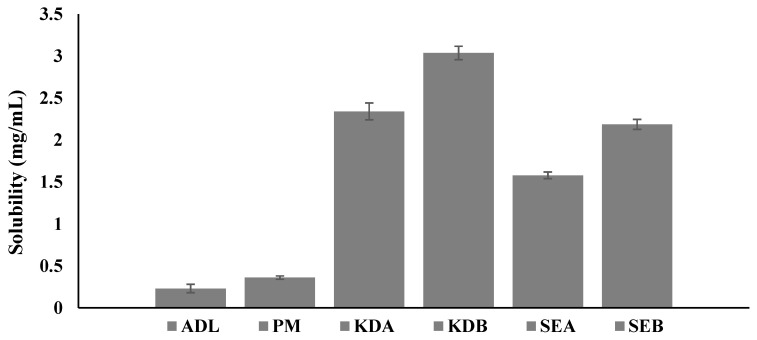
Aqueous solubility of pure ADL and the PM and their binary and ternary complexes. Arbidol (ADL); physical mixture (PM); binary kneaded ADL/β-CD (KDA); ternary kneaded ADL/β-CD with 1% poloxamer 188 (KDB); binary solvent evaporated ADL/β-CD (SEA); ternary solvent evaporated ADL/β-CD with 1% poloxamer 188 (SEB).

The results of the aqueous solubility of pure ADL and the PM and their binary and ternary complexes are shown in Figure 2. The solubility of the poorly soluble ADL drug was significantly improved when complexed with β-CD in the presence or absence of poloxamer 188. The saturated aqueous solubility of pure ADL was found to be 0.23 mg/mL and it was enhanced significantly to be 2.34 mg/mL in the ternary complex (ADL/β-CD with 1% poloxamer 188) prepared by the kneading method, probably due to the surfactant action of poloxamer 188 and the kneading method of preparation [22].

### 2.3. Differential Scanning Calorimetry (DSC)

**Figure 3:**
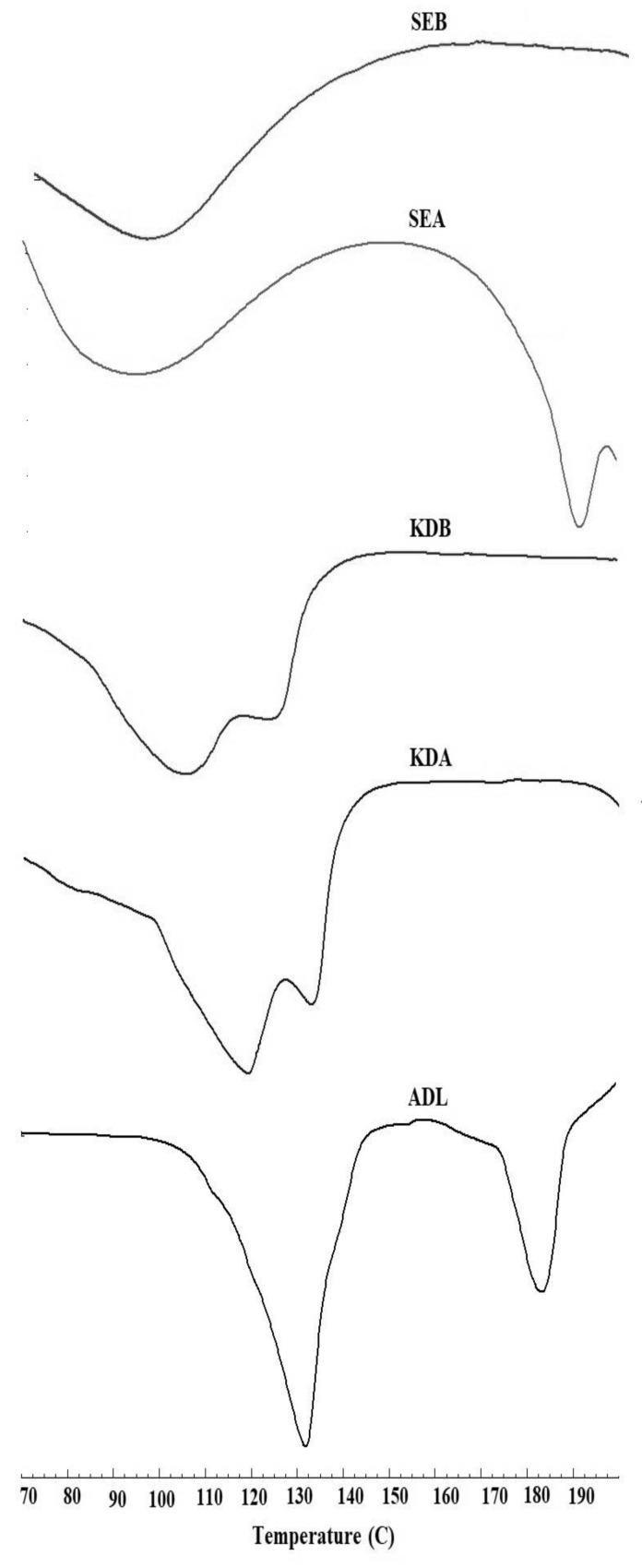
Comparative DSC spectra of pure ADL and the binary and ternary complexes. Arbidol (ADL); binary kneaded ADL/β-CD (KDA); ternary kneaded ADL/β-CD with 1% poloxamer 188 (KDB); binary solvent evaporated ADL/β-CD (SEA); ternary solvent evaporated ADL/β-CD with 1% poloxamer 188 (SEB).

The results of the DSC curve are presented in Figure 3. A broad endothermic peak at 135 °C was observed due to the loss of water and the melting broad endotherm of ADL was found at 185 °C [13]. A dual endothermic peak could be seen near 110–130 °C in the kneaded binary ADL/β-CD (KDA) and ternary ADL/β-CD with 1% poloxamer 188 (KDB) complexes, probably due to the fusion of β-CD and water loss. As shown in Figure 2, the DSC curve of the binary ADL/β-CD and ternary ADL/β-CD with 1% poloxamer 188 prepared by the kneading (KD) and solvent evaporation (SE) method showed a diminished or an absence of an ADL peak indicating encapsulation inside the β-CD cavity. A complete absence of endothermic peaks could be observed in the ternary complex (ADL/β-CD with 1% poloxamer 188) prepared by the KD method. This clearly suggested that a successful inclusion complexation occurred [23].

### 2.4. Fourier Transform Infrared Spectroscopy (FTIR)

**Figure 4:**
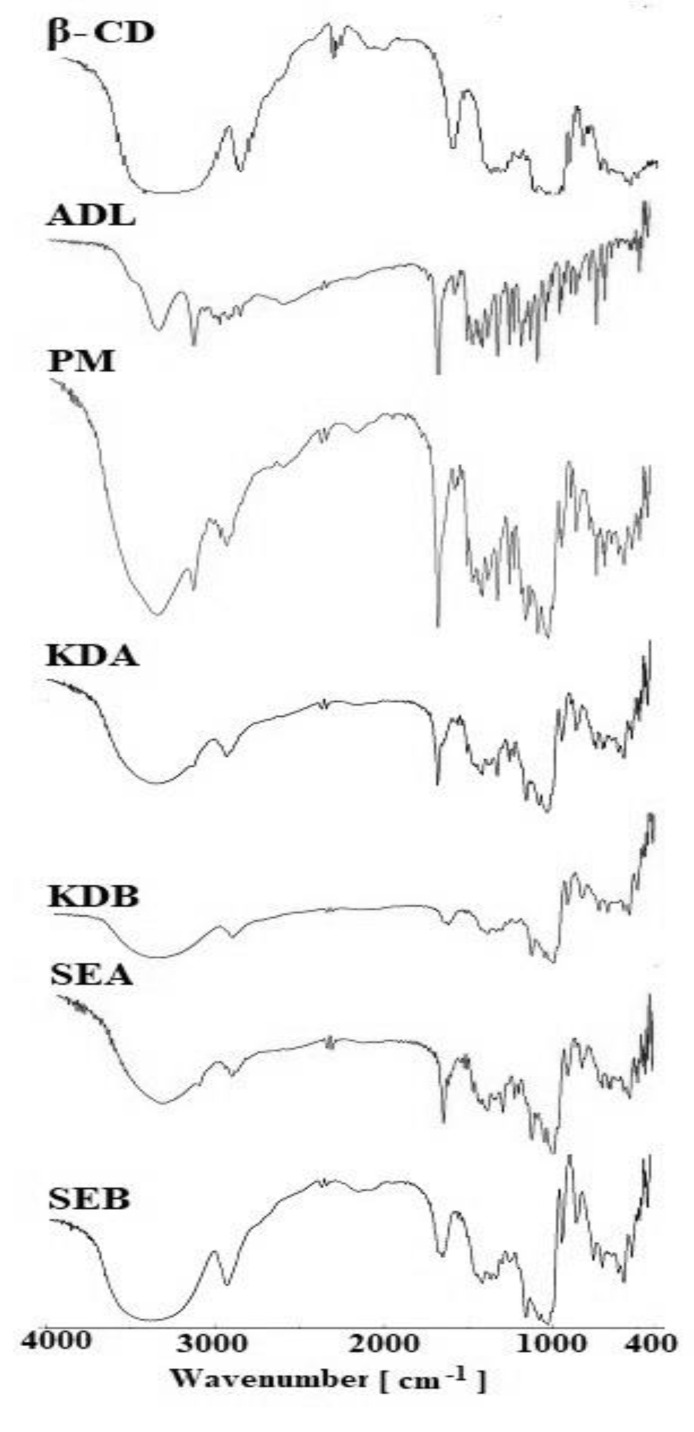
Comparative FTIR spectra of pure ADL and the binary and ternary complexes. Arbidol (ADL); physical mixture (PM); binary kneaded ADL/β-CD (KDA); ternary kneaded ADL/β-CD with 1% poloxamer 188 (KDB); binary solvent evaporated ADL/β-CD (SEA); ternary solvent evaporated ADL/β-CD with 1% poloxamer 188 (SEB).

The results of the FTIR spectra of ADL and its inclusion complexes (PM, KDA, KDB, SEA and SEB) are shown in Figure 4. The FTIR spectra of pure ADL showed a peak at 3327 cm^−1^ for O-H stretching vibrations, 3100 cm^−1^ for aromatic C-H stretching vibrations and 1680 cm^−1^ for C=H stretching vibrations, confirming the purity of the ADL drug [12]. The FTIR spectra β-CD showed characteristic peaks at 3341 cm^−1^ for O-H stretching vibrations and 2934 cm^−1^ for C-H stretching vibrations and other peaks at 1657 cm^−1^, 1278 cm^−1^, 1042 cm^−1^ and 863 cm^−1^ assigned for the presence of H-OH stretching, C-O stretching, C-O-C stretching for the glucose unit and C-O-C stretching vibrations for the ring β-CD. Compared with pure ADL, the binary ADL/β-CD and ternary ADL/β-CD with 1% poloxamer 188 complexes showed the absence of or diminished sharp ADL peaks; other sharp peaks belonging to β-CD could be seen in the spectra of the complexes. However, the ternary ADL/β-CD with 1% poloxamer 188 complex prepared by the kneading technique showed almost a complete disappearance of typical peaks of ADL. Therefore, these results confirmed the complexation of ADL inside the cavity of β-CD.

### 2.5. Powder X-ray Diffraction (PXRD) Studies

**Figure 5:**
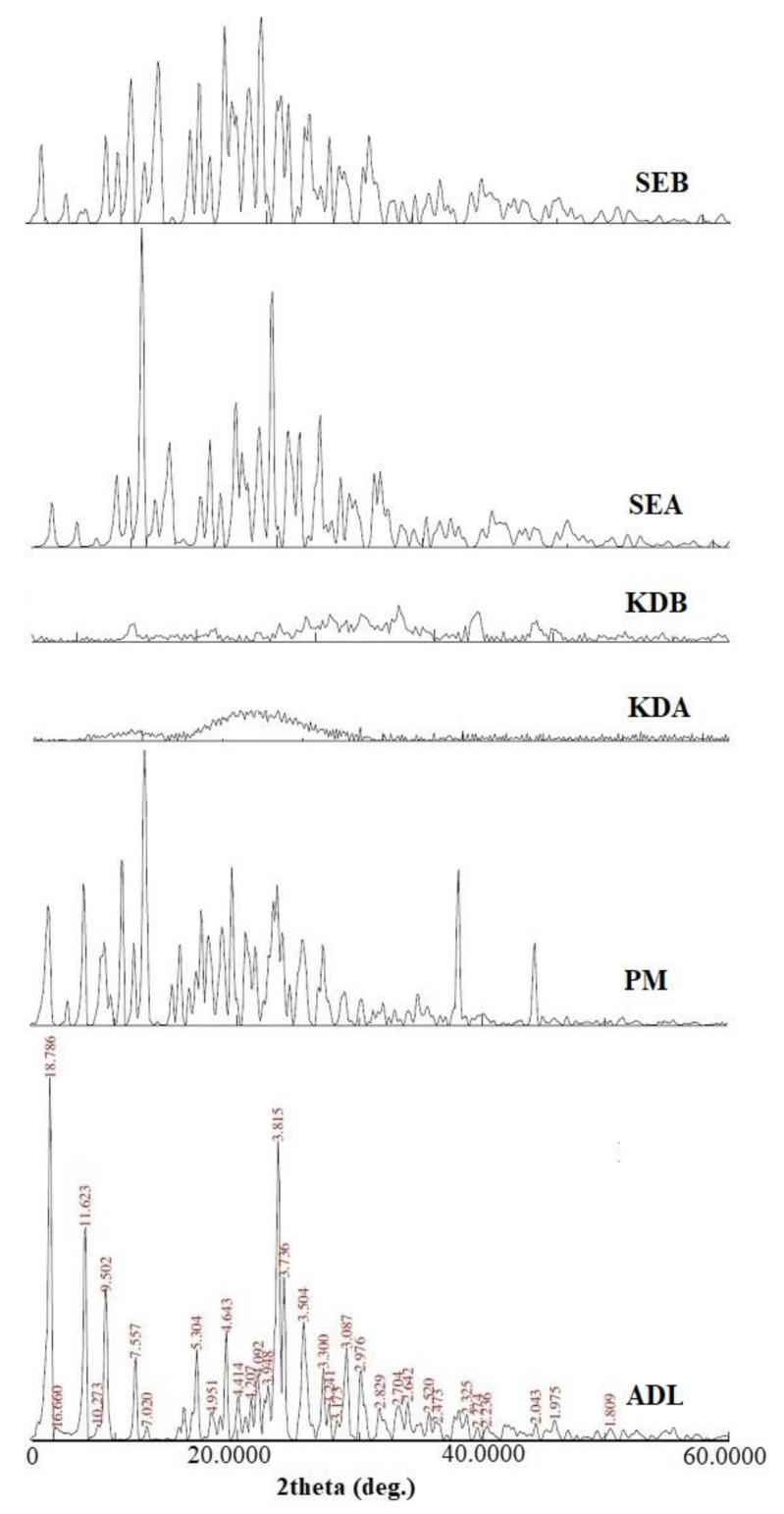
Comparative PXRD spectra of pure ADL, the PM and their binary and ternary complexes. Physical mixture (PM); binary kneaded ADL/β-CD (KDA); ternary kneaded ADL/β-CD with 1% poloxamer 188 (KDB); binary solvent evaporated ADL/β-CD (SEA); ternary solvent evaporated ADL/β-CD with 1% poloxamer 188 (SEB).

The PXRD pattern of ADL, the physical mixture (PM) and the binary and ternary complexes are shown in Figure 5. A few sharp peaks of ADL were found at the diffraction angle of 2θ, 4.7, 7.6, 9.3, 11.7, 16.7, 19.1, 21.7, 23.3, 23.8, 25.4, 27.0, 28.9 and 30.0 that confirmed the crystalline nature of ADL. The PXRD pattern of the PM showed identical peaks as pure ADL suggesting no complexation. The binary (KDA and SEA) and ternary (KDB and SEB) inclusion complexes exhibited a few new diffraction peaks. The sharp peak intensity of pure ADL reduced or disappeared due to the reduction of crystallinity, confirming its successful complexation. The XRD pattern of the ternary complex KDB showed the complete absence of ADL peaks suggesting the complete drug inclusion in the β-CD cavity.

### 2.6. In Vitro Dissolution Studies

**Figure 6:**
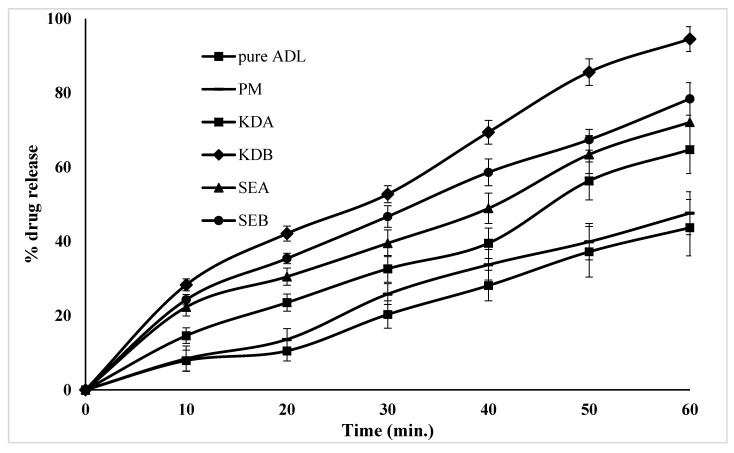
In vitro release profile of pure ADL, the PM and their binary and ternary complexes. Arbidol (ADL); physical mixture (PM); binary kneaded ADL/β-CD (KDA); ternary kneaded ADL/β-CD with 1% poloxamer 188 (KDB); binary solvent evaporated ADL/β-CD (SEA); ternary solvent evaporated ADL/β-CD with 1% poloxamer 188 (SEB).

A comparative release profile of pure ADL, the PM and their binary (KDA, SEA) and ternary (KDB, SEB) complexes measured at different time intervals (10, 20, 30, 40, 50 and 60 min) are shown in Figure 6. Although the % release of pure ADL was increased time dependently, it was found to be only 43.7% after 1 h. However, the % release of ADL was enhanced by 47.6%, 64.7%, 94.5%, 72.1% and 78.4% after 1 h from the PM, KDA, KDB, SEA and SEB, respectively. The maximum enhancement in the dissolution of ADL was found in the ternary kneaded inclusion complex (KDB) probably due to the presence of the surfactant, poloxamer 188, and the process of kneading. A lack of crystallinity (amorphization), increased wettability and dispersibility and particle size reduction are considered to be the important factors for dissolution enhancement in the case of the KDB complex compared with the SEB complexes. Moreover, the dry mixing of a hydrophobic drug with a hydrophilic carrier (poloxamer 188) may result in increasing the surface available for dissolution by reducing the interfacial tension between the hydrophobic drug and the dissolution media.

### 2.7. Morphology

**Figure 7:**
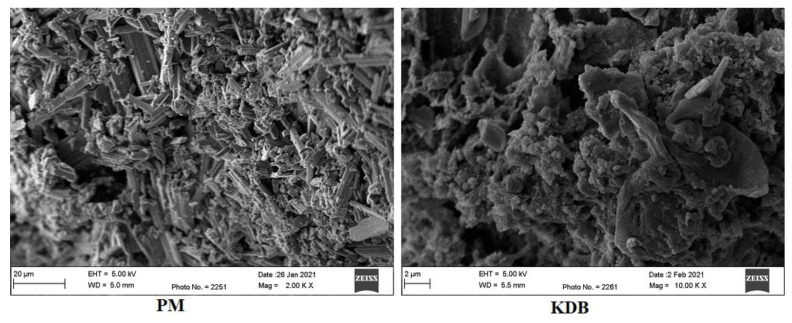
SEM images of the physical mixture (PM) and the optimized kneaded ternary complex (KDB).

The SEM images of the PM and KDB are shown in Figure 7. The crystals of the ADL drug particles could be seen in the images of the PM, suggesting no complexation. However, a poor crystal structure and a lack of crystal structures in the KDB images indicated an amorphous complex formation, which also showed faster dissolution compared with the physical mixture. 

### 2.8. D and 2D ^1^HNMR Studies

**Figure 8:**
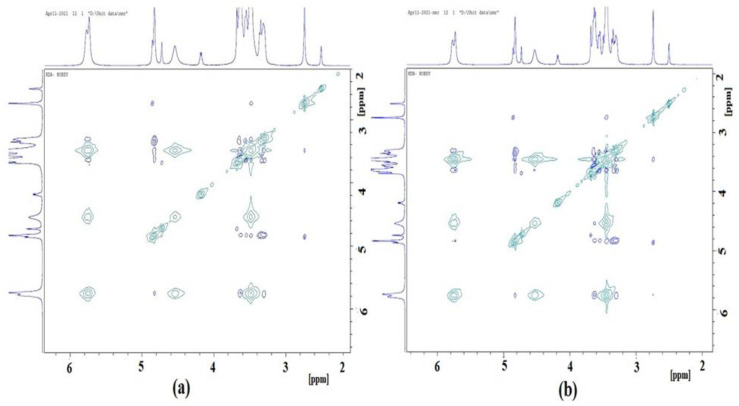
2D ROSEY NMR spectra of (**a**) the kneaded binary complex ADL/β-CD (KDA); (**b**) the optimized kneaded ternary complex ADL/β-CD with 1% poloxamer 188 (KDB).

The formation of the inclusion complexes of ADL into the β-CD cavity with and without 1% poloxamer 188 was evidenced by chemical shift changes detected in ^1^H NMR studies. A significant upfield chemical shift was observed when ADL was complexed with β-CD (KDA) and β-CD with 1% poloxamer 188 (KDB). The H3 and H5 (present in the inner surface of β-CD) showed a maximum CIS in both the KDA and KDB complexes. The higher CIS at H3 (−0.030 and −0.041) and H5 (−0.020 and −0.064) signals were observed in the KDA and KDB complexes, respectively, which suggested the inclusion of ADL inside the cavity of β-CD [24,25]. The 2D ROSEY NMR spectra of the KDA and KDB complexes are shown in Figure 8. The cross signals of NMR were observed, which were an indication of ADL inclusion in the β-CD cavity [26]. 

### 2.9. Pharmacokinetic Studies

**Figure 9:**
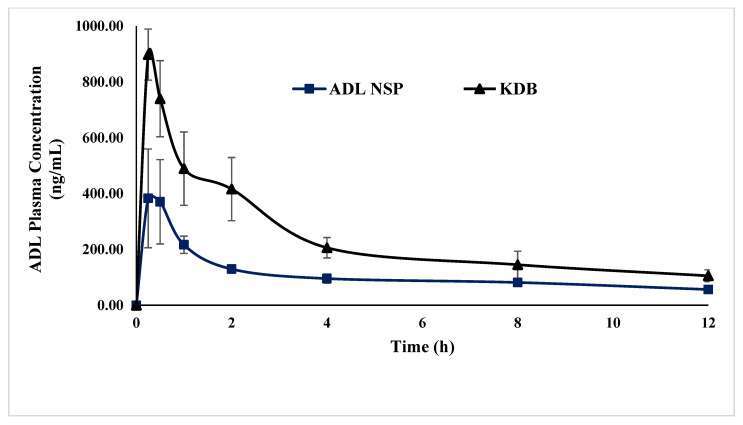
Plasma concertation time profile of the ADL normal suspension (ADL NSP) and ternary kneaded ADL/β-CD with 1% poloxamer 188 (KDB) after the oral administration of 20 mg/kg in rats (*n* = 6 each).

The pharmacokinetic results of a normal ADL suspension and the optimized ternary complex (KDB) are presented in Table 2. The mean plasma concentration versus time profile is presented in Figure 9. The Cmax, AUC0-∞ and T1/2 values achieved in rats after the oral administration of 20 mg/kg of ADL normal suspension were comparable with the results obtained in healthy human volunteers after the oral administration of 200 mg tablets [27]. However, the Cmax, (*p* < 0.05), AUC0-T and AUC0-∞ (*p* < 0.01) were significantly higher in the case of the ADL/β-CD with 1% poloxamer 188 formulation (KDB) compared with the normal suspension of ADL. Therefore, the relative bioavailability was 2.17 fold higher with the optimized ternary complex (KDB). However, the Kel and T1/2 (h) values were not statistically different between these two formulations, indicating that the bioavailability enhancement was due to the rapid absorption of the novel formulation. A good relation between the in vitro release profile and the apparent absorption in the case of the KDB formulation suggested that the rate determining process was the in vivo dissolution of ADL. The MRT value of the optimized ternary complex (KDB) was higher compared with the normal suspension indicating a higher retention of the novel formulation in the body.

## 3. Materials and Methods

### 3.1. Chemicals and Reagents

ADL was purchased from the Beijing Mesochem Technology Co. Ltd. (Beijing, China). β-CD and Pluronic^®^ F-68 (non-proprietary name: poloxamer 188) were obtained from Sigma–Aldrich Pvt. Ltd. (St. Louis, MO, USA). All reagents were of analytical reagent grade and were used without further purification. Deionized water was used for the preparation of aqueous samples. 

### 3.2. Phase Solubility Studies for ADL in Binary and Ternary Systems

Phase solubility studies of ADL/β-CD and ADL/β-CD with 1% poloxamer 188 were used to evaluate the complexation [21]. In this study, excess amounts of ADL were added to 10 mL of distilled water containing increment amounts of β-CD and β-CD with 1% poloxamer 188 (5–30 mM). The mixed suspensions were shaken at 100 rpm and 37 °C for 72 h in a water bath. The resultant suspension was filtered using a 0.45 µm membrane filter and then analyzed by UV at 258 nm [28]. The association strengths of ADL/β-CD and ADL/β-CD with 1% poloxamer 188 were calculated in terms of the apparent stability constant (*Ks*) using the following equation:

$$K_{s}=\frac{Slope}{S_{{}^{\circ}}\times \left(1-slope\right)}$$

where *S_o_* was the intrinsic solubility of arbidol in water and Slope was the slope of the phase solubility diagram.

The complexation efficiency (*CE*) was the ratio of the complex formed to free the β-CD concentration, which was an excellent method of calculating the solubilizing effect of β-CD. The *CE* was estimated using the following formula.

$$CE=\frac{Slope}{1-Slope}.$$

### 3.3. Preparation of the Complexes

#### 3.3.1. Preparation of the Physical Mixture (PM)

The inclusion complex of ADL/β-CD (1:1) was prepared by mixing in a clean glass mortar and pestle.

#### 3.3.2. Preparation of the Complexes by the Kneading (KD) Method

**Figure 10:**
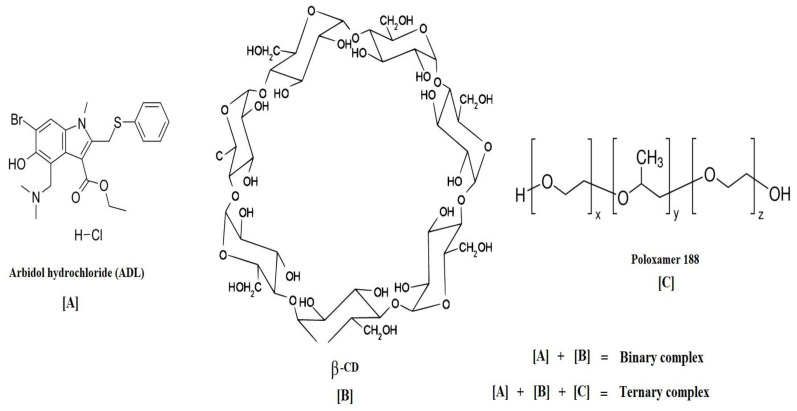
Chemical structure of arbidol hydrochloride, β-cyclodextrin and poloxamer 188.

The inclusion complexes containing ADL/β-CD (1:1) were established in preliminary phase solubility studies. The inclusion complexes of ADL/β-CD and ADL/β-CD with 1% poloxamer 188 in a 1:1 molar ratio were prepared by utilizing kneading. The binary complex of ADL/β-CD in a molar ratio (1:1) was prepared by mixing one mole of ADL and one mole of β-CD in a mortar followed by the addition of about 6 mL of a water-ethanol solution (50%, *v*/*v*). The mixture was continuously kneaded for 30 min until a thick paste was formed, which was evaporated at 50 °C for 4 h. The solid powder was then crushed, passed through a sieve and stored in airtight containers. The same procedure was followed to prepare the ternary complex of ADL /β-CD as described above but 1% poloxamer 188 was kneaded with the ADL /β-CD complex (Figure 10).

#### 3.3.3. Preparation of the Complexes by the Solvent Evaporation (SE) Method

The inclusion complexes of ADL/β-CD and ADL/β-CD with 1% poloxamer 188 were prepared by the SE method. Briefly, accurately weighed amounts of ADL and β-CD were dissolved in 40 mL of ethanol:water (1:2). The obtained solution was then dried on a rota evaporator at 50 rpm for 4 h (Buchi Rotavapor R-215). The same procedure was followed to prepare the ternary complex of ADL/β-CD but 1% poloxamer 188 was dissolved with the ADL/β-CD complex (Figure 10).

### 3.4. Characterization of the Complexes

#### 3.4.1. Aqueous Solubility Determination of the Solid Complexes

Excess amounts of pure ADL, the PM and their binary and ternary complexes were kept in conical flasks containing 10 mL of distilled water and stirred on a thermostatic mechanical shaker (LBS-030S, Lab Tech, Korea) at 25 °C for 72 h [29]. All samples were inspected for saturation after 2, 4, 6, 12, 24, 48 and 72 h. After 72 h, each sample suspension was filtered through a millipore filter (0.45 μm) and analyzed for drug content by UV at 258 nm [28].

#### 3.4.2. DSC Studies

The DSC thermal curves of the prepared complexes were recorded using a DSC instrument (Scinco, DSC N-650, Seoul, Korea). The samples (5 mg) were crimped in aluminum pans by applying pressure and the pan was placed in the sample holder of the instrument and heated at a temperature of 50–200 °C at a heating rate of 10 °C/min. The DSC instrument was purged with nitrogen gas during the analysis [30,31].

#### 3.4.3. FTIR Studies

The FTIR spectra of pure ADL, the PM and their binary and ternary complexes prepared by the KD and SE methods were recorded using an FTIR spectrometer (Jasco FTIR Spectrophotometer, Tokyo Japan). The samples were pressed into transparent pellets by diluting with crystalline potassium bromide. The FTIR spectra were interpreted using spectra manager software [30,31].

#### 3.4.4. PXRD Studies

The PXRD pattern of pure ADL, the PM and their binary (KDA, SEA) and ternary (KDB, SEB) complexes were recorded by an Ultima IV diffractometer (Rigaku Inc. Tokyo, Japan at the College of Pharmacy, Prince Sattam Bin Abdulaziz University) in the range of 0–80° (2θ) at a scan rate of 0.5°/min. The recorded PXRD patterns of each sample were evaluated for inclusion complexation [32].

### 3.5. In Vitro Dissolution Studies

In vitro dissolution studies were performed to compare the release profile of the physical mixture (PM) with the binary kneaded (KDA), solvent evaporated (SEA), ternary kneaded (KDB) and solvent evaporated (SEB) inclusion complexes with pure ADL. The pure ADL and complexes (equivalent to 40 mg of ADL) were filled into hard gelatin capsules and each filled capsule was dipped into a basket containing 900 mL of 0.1N HCl (pH 1.2). The stirring speed was set at 50 rpm at a temperature of 37 ± 1 °C. The samples were withdrawn at different time intervals and analyzed by UV at 258 nm [28].

### 3.6. Morphology

The surface morphology of the physical mixture (PM) and the optimized kneaded ternary inclusion complex (KDB) were studied under the scanning electron microscopy (SEM) (SEM, Ultra Plus, Zeiss, Oberkochen, Germany). The samples were vortexed for 5 min after a suitable dilution with methanol. One drop of solution was spread onto a slide and kept for drying before the slide was examined for morphology [32].

### 3.7. 1D and 2D 1HNMR Studies

1HNMR spectra were recorded using a DMSO-d6 solvent and an UltraShield Plus 500 MHz Bruker spectrometer operating at 500 MHz for proton atoms at the Prince Sattam Bin Abdulaziz University and 2D NMR (ROSEY) spectra were acquired using the Bruker library. Briefly, the pure ADL drug, β-CD, the binary kneaded (KDA) and ternary kneaded (KDB) complexes were dissolved in DMSO-d6 and filtered in a 5 mm glass capillary tube and then the 1HNMR spectra were recorded. The chemical shift values were measured in δ (ppm). The complexation induced shifts (CIS = δcomplexed β-CD = δfree β-CD) were also calculated to discover the inclusion of ADL into the β-CD cavity.

### 3.8. Bioanalytical Method Conditions

The quantitative analysis of ADL was performed on an Acquity UPLC system connected with a triple quadruple (TQD) detector (Waters Corp., Milford, MA, USA) using ibrutinib as an internal standard (IS). The electrospray ionization in a positive mode was used for the sample ionization in a multiple reaction monitoring (MRM) mode. The precursor to the production ion transition of 477.05 > 279.02 and 441.16 > 84.4 were used in the MRM mode for the detection of ADL and the IS. The optimized mass spectrometry conditions were capillary voltage (0.5 kV), source temperature (150 °C), desolvation temperature (350 °C), desolvation gas (nitrogen) flow rate (600 L/h) and collision gas (argon) flow rate (0.13 mL/min). The cone voltage and collision energy of ADL and the IS were optimized to 30 and 48 V and 36 and 40 eV, respectively [33].

The chromatographic separation was performed on an Acquity UPLC BEH column (2.1 × 100; 1.7 μm). The mobile phase comprising of acetonitrile and 15 mM ammonium acetate in a composition ratio of 80:20 (*v*/*v*) was used at a flow rate of 0.3 mL/min. The total run time of the analysis was 2.5 min only. The calibration curves were linear between the concentration ranges of 1.32–625 ng/mL with a lower limit of quantification (LOQ) of 1.32 ng/mL. All of the validation parameters were within the acceptable limits as per the guidelines for a bioanalytical method validation.

Prior to the analysis, samples were thawed at room temperature and 150 µL aliquot of plasma was transferred to 2mL capacity of an Eppendorf tube. A total of 20 µL of the IS (250 ng/mL) was then spiked into each tube except the blank samples and vortex-mixed properly. One mL of ethyl acetate was transferred to each tube followed by cold centrifugation at 4500× *g* for 10 min. The supernatant organic layer was transferred to a 1.5 mL capacity Eppendorf tube and all samples were transferred to the sample concentrator for drying. After the drying step, the remaining residue was reconstituted by neat acetonitrile and 5 µL was injected into a UPLC-MS/MS system for the analysis.

### 3.9. Pharmacokinetic Studies 

The pharmacokinetic study of the newly developed and the optimized ternary complex (KDB) was compared with the normal suspension of ADL in male wistar albino rats. The experimental protocol was reviewed and approved by the Research Ethics Committee (Approval number: BERC-003-03-21) and all applicable international guidelines including the Animal Care and Use Committee of the Prince Sattam Bin Abdulaziz University were followed. The twelve rats (weight 180–210 gm) were received from the Animal Care Centre, College of Pharmacy, Prince Sattam Bin Abdulaziz University, Alkharj, Saudi Arabia and were kept under the recommended conditions with access to food and water ad libitum. Before the experiment, all animals were allowed to acclimatize to the normal conditions for one week. The rats were divided into two groups (*n* = 6 each); one group received a normal suspension of pure ADL prepared in 0.5% *w*/*v* carboxyl methyl cellulose and the other group received the optimized ternary complex (KDB) (20 mg/Kg). The blood samples (0.5 mL) were collected in a pre-heparinized tube at different time intervals (predose, 0.25, 0.5, 1, 2, 4, 8 and 12 h). All blood samples were centrifuged at 4500× *g* for 5 min and plasma was harvested and placed in a deep freezer maintained at 80 ± 10 °C until further UPLC-MS/MS analysis.

The pharmacokinetic parameters were calculated using WinNonlin software (Pharsight Co., Mountain View, CA) and the results were expressed as a mean ± standard deviation (SD). A non-compartmental pharmacokinetic model was used to calculate the maximum concentration (Cmax) and the time to reach the maximum concentration (Tmax), the AUC from 0 to t (AUC0–12) and 0–inf (AUC0-∞), the elimination rate constant (kz), the half-life (T½) and the mean residence time (MRT). An unpaired t-test was used to compare the results between the normal ADL suspension and ADL/β-CD with 1% poloxamer 188 considering *p* < 0.05 as statistically significant.

## 4. Conclusions

In the present study, the complexation efficiency of ADL with β-CD was investigated in the presence and absence of poloxamer 188. Both the binary ADL/β-CD and the ternary ADL/β-CD with 1% poloxamer 188 complexes were prepared by kneading and solvent evaporation methods and were further characterized by aqueous solubility, FTIR, PXRD, DSC, SEM, 1HNMR and in vitro studies. The solubility and release of ADL was remarkably improved (13.1 fold) in the kneaded ternary complex ADL/β-CD with 1% poloxamer 188 (KDB). The pharmacokinetic study revealed that the ADL formulation composed of KDB showed a significant improvement in relative bioavailability (2.71 fold) in comparison with the pure ADL suspension. Thus, the above study demonstrated that the solubilization and bioavailability of ADL can be improved by the formation of an ADL/β-CD complexation in the presence of a third component, which is poloxamer 188. 

## Figures and Tables

**Table 1:** Complexation parameters of ADL/β-CD and ADL/β-CD with poloxamer 188.

Complexes	Stability Constant (*Ks*)	Complexation Efficiency (CE)
Arbidol/β-CD	550 M^−1^	0.0064
Arbidol/β-CD with 1% Poloxamer 188	2134 M^−1^	0.0250

**Table 2:** Pharmacokinetic parameters of pure ADL and ternary kneaded ADL/β-CD with 1% poloxamer 188 (KDB).

Parameters	ADL NSP (*n* = 6)	(KDB) (*n* = 6)
Cmax(ng/mL)	387 ± 171	897 ± 92 *
Tmax(h)	0.25	0.25
AUC0-T(ng.h/mL)	1317 ± 127	2858 ± 674 **
AUC0-∞ (ng.h/mL)	1838 ± 31	3745 ± 1105 **
Kel(h)	0.11 ± 0.03	0.12 ± 0.02
T1/2 (h)	6.57 ± 1.79	6.04 ± 1.74
MRT (h)	9.35 ± 2.09	7.96 ± 1.82
Relative Bioavailability (%)	100	217

** p < 0.01 * p < 0.05, ADL normal suspension (ADL NSP).

## Data Availability

The datasets used and/or analysed during the current study are available from the corresponding author on reasonable request.
